# The unmasking of *Pneumocystis jiroveci *pneumonia during reversal of immunosuppression: case reports and literature review

**DOI:** 10.1186/1471-2334-4-57

**Published:** 2004-12-09

**Authors:** Alan KL Wu, Vincent CC Cheng, Bone SF Tang, Ivan FN Hung, Rodney A Lee, David S Hui, Kwok Y Yuen

**Affiliations:** 1 Division of Infectious Diseases, Centre of Infection, Queen Mary Hospital, The University of Hong Kong; Hong Kong Special Administrative Region, China; 2 Division of Pulmonary Medicine, Department of Medicine and Therapeutics, Prince of Wales Hospital, The Chinese University of Hong Kong; Hong Kong Special Administrative Region, China

## Abstract

**Background:**

Pneumocystis jiroveci pneumonia (PCP) is an important opportunistic infection among immunosuppressed patients, especially in those infected with human immunodeficiency virus (HIV). The clinical presentation of PCP in immunosuppressed patients have been well-reported in the literature. However, the clinical importance of PCP manifesting in the setting of an immunorestitution disease (IRD), defined as an acute symptomatic or paradoxical deterioration of a (presumably) preexisting infection, which is temporally related to the recovery of the immune system and is due to immunopathological damage associated with the reversal of immunosuppressive processes, has received relatively little attention until recently.

**Case presentation:**

We aim to better define this unique clinical syndrome by reporting two cases of PCP manifesting acutely with respiratory failure during reversal of immunosuppression in non-HIV infected patients, and reviewed the relevant literature. We searched our databases for PCP cases manifesting in the context of IRD according to our predefined case definition, and reviewed the case notes retrospectively. A comprehensive search was performed using the Medline database of the National Library of Medicine for similar cases reported previously in the English literature in October 2003. A total of 28 non-HIV (excluding our present case) and 13 HIV-positive patients with PCP manifesting as immunorestitution disease (IRD) have been reported previously in the literature. During immunorestitution, a consistent rise in the median CD4 lymphocyte count (28/μL to 125/μL), with a concomitant fall in the median HIV viral load (5.5 log_10 _copies/ml to 3.1 log_10 _copies/ml) was observed in HIV-positive patients who developed PCP. A similar upsurge in peripheral lymphocyte count was observed in our patients preceding the development of PCP, as well as in other non-HIV immunosuppressed patients reported in the literature.

**Conclusions:**

PCP manifesting as IRD may be more common than is generally appreciated. Serial monitoring of total lymphocyte or CD4 count could serve as a useful adjunct to facilitate the early diagnosis and pre-emptive treatment of this condition in a wide range of immunosuppressed hosts, especially in the presence of new pulmonary symptoms and/or radiographic abnormalities compatible with the diagnosis.

## Background

*Pneumocystis jiroveci *(Pj) (previously known as *Pneumocystis carinii *f. sp. *hominis*) was first identified as a pathogen in premature infants suffering from interstitial plasma cell pneumonia in European countries during and after World War II, occasionally occurring in epidemics [1-3]. Since then Pneumocystis pneumonia (PCP) had only been reported sporadically in patients with malignancies and solid organ transplantations until the HIV epidemic [4]. The incidence of PCP increased significantly after the emergence of human immunodeficiency virus (HIV) infection. However, with the identification of CD4 T lymphocyte depletion as an independent risk factor for the development of PCP [5], widespread use of antimicrobial prophylaxis [4], and the introduction of highly active antiretroviral therapy (HAART), there has been a steady decline in the incidence of PCP among HIV-infected patients [7,8].

Nevertheless, with the rising number of patients receiving immunosuppressive therapies for malignancies, solid organ transplantations and autoimmune diseases, PCP has been increasingly recognized in non-HIV immunosuppressed hosts [9-15]. For instance, PCP occurs in 3.4% to 43% of solid organ transplant recipients [16], and it is particularly prevalent among those patients who are put on long-term steroids. In a non-HIV immunosuppressed cohort with PCP, the use of steroids was found to be a contributing factor in 87% of patients [17]. In another similar cohort of immunosuppressed patients, steroids had been administered systemically in 90.5% within one month before the diagnosis of PCP. Although a median daily dose equivalent to 30 mg of prednisone was administered in most of these patients prior to the development of PCP, up to 25% had received as little as 16 mg of prednisone daily [18]. Interestingly, PCP has also been reported in patients with endogenous steroid excess due to Cushing's disease [19,20].

Paradoxically, the clinical symptoms of PCP were often unmasked in HIV-negative immunosuppressed patients during the reversal of immunosuppression, often at the time when the dose of steroids was tapered [11,17,21-24], or when the endogenous steroid production was reduced [25-27]. However, serial changes in the absolute lymphocyte count before and during reversal of immunosuppression were not mentioned in these patients. Recently, paradoxical worsening of clinical symptoms and signs of PCP after initiation of HAART has also been reported in HIV-positive patients [28-31]. The onset of clinical deterioration was associated with an upsurge in the CD4 lymphocyte count and a reduction in the HIV viral load [28-31]. Tissue damage is thought to occur as a result of immune reconstitution in HIV-positive patients. Here, we report two cases and review the literature on this topic from the perspective of immunorestitution disease.

## Case presentation

### Case 1

This is a fifty-one year old female patient with history of diabetes mellitus and systemic lupus erythematoses (SLE) complicated by lupus nephritis. Although we have included her case in our previous publication [32], we have not reported her clinical details at that time. She was put on prednisolone 30 mg and azathioprine 100 mg daily since end of June and mid-July 2002, respectively. She was admitted to Queen Mary Hospital on 11^th ^August 2002 for investigation of jaundice. Investigations revealed deranged liver function tests with cholestatic pattern. A diagnosis of drug-induced hepatotoxicity was entertained, and azathioprine was stopped after admission. As her autoimmune disease was under control, her steroid dosage was reduced from 25 mg to 15 mg daily within the next 14 days. Her CXR taken on admission was normal.

Soon after her immunosuppressive therapy was tapered, she developed fever and non-productive cough. A repeat CXR performed on 9^th ^Sept revealed new infiltrate over the left mid-zone, suggestive of pneumonia. She was started on intravenous ceftazidime 1 gram eight hourly and oral clarithromycin 500 mg twice daily. Serial CXR performed three days later showed increasing bilateral pulmonary infiltrates and worsening hypoxemia. There was an upsurge of total lymphocyte count from 0.7 × 10^9^/L (total white cell count 7.2 × 10^9^/L) at the time of admission to 5.6 × 10^9^/L (total white cell count 10.8 × 10^9^/L) at the time of clinical deterioration. Bronchoscopy with transbronchial biopsy performed on the same day revealed *Pneumocystitis jiroveci *by methenamine sliver stain. Workup for other opportunistic pathogens including cytomegalovirus and aspergillus was negative. She was commenced on intravenous pentamidine (4 mg/kg/day) and corticosteroids for severe PCP infection. Despite active treatment she developed progressive respiratory failure and required admission to intensive care unit. She subsequently recovered after a stormy hospital course, and upon discharge from hospital, her total lymphocyte count had returned to her baseline of 0.86 × 10^9^/L.

### Case 2

A thirty-three year old gentleman initially presented to Prince of Wales Hospital with a diagnosis of SLE/dermatomyositis overlap syndrome. He was treated with steroid and hydroxychloroquine 200 mg twice daily since 1997. He had a flare up of disease in May 1998 with active vasculitis and myositis, for which he was put on prednisolone and azthioprine 50 mg and 100 mg daily respectively. Upon reassessment one month later, disease activity was under control, and the dosage of prednisolone was reduced to 45 mg daily.

Twelve day after reducing the immunosuppressive regimen, he was admitted to hospital for treatment of left buttock abscess. The CXR taken on admission was unremarkable. An aspirate of the pus from the lesion grew methicillin-sensitive *staphylococcus aureus*; he was treated with cloxacillin 1 g intravenously every 6 hourly, together with incision and drainage of the buttock abscess. In view of the underlying active pyogenic infection, the steroid dosage was rapidly tapered from 45 mg to 15 mg daily within the next four days. However, he was noted to have persistent fever associated with mild unproductive cough. A repeat chest radiograph showed new infiltrates over the right upper and left lower zones, and he was empirically treated with intravenous ceftazidime 1 gram every 8 hours, cloxacillin 1 gram every 6 hours and netimicin 100 mg every 8 hours. As there was no clinical response after 5 days of treatment, bronchoscopy and bronchoalveolar lavage (BAL) was performed, which was positive for *Pneumocystis jiroveci*. Investigation for the presence of co-existing opportunistic pathogens such as cytomegalovirus and aspergillus was negative. On the day after bronchoscopy, he was commenced on intravenous cotrimoxazole 1.3 grams every 6 hours. He remained stable initially with fever on downward trend. However, on the 3^rd ^day of treatment, he developed sudden desaturation with resurgence of high fever, and required supplemental oxygen therapy. Repeat chest radiograph showed increased perihilar hazziness in both lung fields. There was also an upsurge of total lymphocyte count from 0.6 × 10^9^/L (total white cell count 11.2 × 10^9^/L) on admission, to 1.3 × 10^9^/L (total white cell count 10.4 × 10^9^/L) at the time of clinical deterioration. He was treated with high dose prednisolone (80 mg daily), and his condition improved promptly afterwards. He was subsequently discharged, and on follow up at the clinic one month later, his total lymphocyte count had returned to his baseline level of 0.6 × 10^9^/L.

Immunorestitution disease (IRD) has been described in both HIV and non-HIV immunosuppressed hosts previously [27-31]. In the setting of PCP, it is defined as an acute symptomatic presentation of the disease that is related temporally to the recovery of the immune system, associated with reversal of immunosuppressive processes such as reduction in the dosage of corticosteroids and/or cytotoxic agents or a reduction of HIV viral load due to HAART, which results in the development of immunopathological damage. The preexisting microbial infection could be either asymptomatic or mildly symptomatic. Using this case definition, we attempted to review the English literature for other reported cases of PCP manifesting as IRD. The English-language literature (1966 – 2003) was searched in the Medline database of the National Library of Medicine in October 2003. The keywords "*Pneumocystis carinii*", "*Pneumocystis jiroveci*", "HIV", immunosuppression", "immunosuppressive", "steroid", and "corticosteroid" were used to select cases. All the case reports and case series with clinical details were included in this study if they fulfilled the above definition of IRD. When appropriate, the cited bibliographies were also retrieved for further analysis. As for statistical analysis, we used the Wilcoxon Signed Rank test, a non-parametric test for comparing paired samples, to analyze the serial changes in lymphocyte counts and HIV viral loads before and during the development of IRD. A two-tailed p-value of less than 0.05 was considered significant. All statistical analyses were performed using SPSS version 11.5 for Windows.

Including our present case, a total of 29 cases of PCP in non-HIV immunosuppressed hosts fulfilling our definition of IRD have been reported in the literature (table 1) [22-27,32]. There were altogether 13 males and 8 females, with a median age of 38 years (range 2 to 75 years). The age and sex were not mentioned in 8 cases. Their underlying immunosuppressive conditions included solid organ tumours (13 cases), haematological diseases (8), autoimmune diseases (4), endogenous Cushing's disease (3), and a solid organ transplant recipient (1). All patients had received steroids or had excessive endogenous steroid production, whereas 18 (62.1%) of them had concomitant cytotoxic therapy for the underlying diseases. The median duration between steroid tapering and clinical manifestations of PCP was 21 days (range 1 to 83 days). Steroids were completely withdrawn at a median of 7.5 days (range 1 to 21 days) before the onset of symptoms in eight patients. Serial lymphocyte counts were only available in eight patients. An upsurge of the absolute lymphocyte counts was observed from the time of reduction of immunosuppression (median 300/μL, range 290 to 600/μL at baseline) to the time of occurrence of IRD (median 1200/μL, range 600 to 5620/μL); the median increase in total lymphocyte count was 800/μL, with a range of 300 to 4880/μL. Comparing the lymphocyte counts before and after reversal of immunosuppressive therapy, the difference was statistically significant (Wilcoxon Signed Rank Test for paired samples; p = 0.012). In addition to our patient, reintroduction or increasing doses of steroids were required in 7 (53.8%) of 13 patients in the acute management of PCP in the literature, at the time when they developed clinical deterioration during antimicrobial therapy [24-27]. Seven (53.8%) of 13 cases had respiratory failure requiring mechanical ventilation. Among these 29 cases, 13 (44.8%) subsequently died of PCP.

Among HIV-positive patients, 13 cases with newly diagnosed PCP were reported in the literature, in which IRD occurred shortly after the introduction of HAART (table 2) [28-31]. Seven (53.8%) out of 13 cases received steroids as adjunctive therapy in addition to antimicrobials. HAART was given in all cases at a median 18 days (range 1 to 35 days) after the initiation of treatment for PCP. During IRD, recurrence of fever (100%), dyspnoea (100%), and paradoxical worsening of pulmonary infiltrates (58.3%) were observed in these patients [28-31]. IRD occurred at a median of 14 days (range 5 to 17 days) after HAART. An upsurge of the CD4 lymphocyte count was observed before (median 28/μL, range 4 to 290/μL) and during IRD (median 125/μL, range 30 to 564/μL); this was associated with a concomitant reduction of the median HIV viral load from 5.5 log_10 _copies/ml (range 5.0 to 5.9 log_10 _copies/ml) to 3.1 log_10 _copies/ml (range 2.9 to 4.5 log_10 _copies/ml) before and during IRD respectively, and the differences observed in both the CD4 counts and viral loads before and during IRD reached statistical significance (Wilcoxon Signed Rank Test for related samples; p = 0.001 and 0.017, respectively). Antimicrobials, steroids, or both for PCP were reintroduced for IRD in 4, 1, and 6 cases respectively. Only 2 cases were treated conservatively. One case required mechanical ventilation for severe respiratory distress. None of the patients died.

PCP manifesting as a form of IRD is not a rare phenomenon. As shown in our previous study, it happens in 7 out of 10 (70%) of HIV-negative immunosuppressed hosts infected with Pj [32]. However, the diagnosis of PCP is usually delayed in this group of patients because of atypical presentation. In this clinical setting, PCP manifesting as IRD often runs an acute and fulminant course, with nonspecific lesions on chest radiographs, and high absolute lymphocyte counts [32]. In our own reported series, despite the administration of steroid therapy to suppress the immunopathological damage, more than 80% of patients developed acute respiratory failure and required mechanical ventilation. Patients who developed PCP during reversal of immunosuppressive therapy in our series tended to be older, and this might partially explain the increased mortality observed in this group [32].

Rapid reduction of immunosuppressive therapy such as steroids has been implicated as a predisposing factor for the development of PCP in HIV-negative patients [11,17,23,24]. In one study, PCP occurred in 79 (70%) of 113 patients during steroid tapering [17]. Another study suggested that 8 (72.2%) out of 11 episodes of PCP developed when steroid therapy was tapered [23]. A subsequent study also demonstrated that 43% of patients had a rapid reduction of steroid dosing before the clinical manifestations of PCP [11]. A similar experience was reported in children, and 17 (89.5%) out of 19 children were diagnosed to have PCP during steroid tapering according to a previous report [21]. Another series revealed that 7 of 11 patients experienced acutely symptomatic PCP when the dose of steroids was decreased or terminated 5 days to 3 weeks before the diagnosis of PCP [22]. However, all these cases were not analyzed from a perspective of IRD. Serial changes of the absolute lymphocyte counts or their subsets were either not noted or reported [11,17,21-24]. Hence we have not included these cases for further analysis in this review.

Among HIV positive patients, PCP manifesting acutely during the initiation anti-retroviral therapy is a well-recognized phenomenon. The underlying immunopathological nature of this condition, which is reminiscent to IRD occurring in non-HIV infected patients, has been confirmed by histological examination of the lungs and transbronchial biopsy specimens, which demonstrated mixed inflammatory infiltrates including macrophages, neutrophils, lymphocytes, and plasma cells. Almost all infiltrating lymphocytes found in the tissues were of the T cell lineage, shown by immunophenotyping to be predominantly CD4 and CD8 cells [28]. In another study [30], the BAL fluid obtained from one of six patients with an IRD-type presentation of PCP was analyzed. Infiltration of predominantly CD4 and CD8 lymphocytes with the proliferative marker (Ki67) and perforin-positive cell were seen in the BAL specimen. Therefore, it is likely that the phagocytosed Pj is presented by alveolar macrophage to T cells, which trigger the inflammatory response [30].

In our own experience, as well as from the review of published literature, it appears that a surge of absolute lymphocyte count, especially the CD4 lymphocyte count in HIV-positive patients, could potentially act as a surrogate marker for immunopathological damage during IRD in both HIV-negative and HIV-positive patients. In our recent publication [32], 7 out of 10 non-HIV immunosuppressed patients demonstrated a consistent rise in the absolute lymphocyte count during tapering of immunosuppression prior to the onset of symptomatic PCP. In this group of patients, the surge in lymphocyte count is likely the result of withdrawal of lymphocytotoxic immunosuppressants such as corticosteroids. Similarly, a rising trend of the CD4 lymphocyte count, consistent with immune reconstitution after HAART, was also observed in 13 HIV-positive cases before and during the development of symptomatic PCP [28-31]. In fact, an upsurge in the absolute lymphocyte count has been shown to be a marker of IRD in our previous publications involving viral and tuberculous infections [33-36]. However, it must be emphasized that the number of circulating lymphocytes may not always correlate with their number in the affected tissues or their in vivo functional activity. This can be exemplified by a case of PCP occurring during steroid withdrawal, in which the lymphocyte counts surged to a very high level and then rapidly dropped to a low level within one day. The migration of lymphocytes from the circulation to tissue might explain this rapid drop in lymphocyte count and the resulting immunopathological damage [27]. In the future, further studies on lymphocyte subsets and cytokine profiles of susceptible hosts during the development of IRD should be performed to elucidate the underlying immunopathological mechanisms behind this interesting phenomenon.

From the result of this review, it appears that HIV-positive patients with PCP are at risk of clinical deterioration due to IRD if HAART therapy is started within 1 to 2 weeks after the initiation of treatment for PCP (table 2). With a better understanding of the pathogenetic mechanisms resulting in IRD, we may be able to prevent the occurrence of IRD by delaying the initiation of HAART in HIV-positive patients with PCP. However, in non-HIV immunosuppressed patients, it is even more important to recognize the atypical presentations of PCP in the context of IRD. Since the clinical and/or radiological features alone may not be sufficient for diagnosis, analysis of serial changes in lymphocyte counts in patients undergoing a reduction of immunosuppression can alert the clinician to the possibility of IRD due to occult pathogens such as Pj. To prevent IRD in non-HIV immunosuppressed patients, the use of prophylactic antibiotics against Pj to reduce the microbial load in selected patients remains an important issue. Recently, a multi-center study showed that the CD4 lymphocyte count may be a useful marker to monitor the risk of development of PCP in non-HIV immunosuppressed hosts [37], and patients with low CD4 lymphocyte counts of less than 300 or 400 may require prophylaxis. In fact, asymptomatic colonization of Pj has been demonstrated in HIV-negative patients when the CD4 lymphocyte count was less than 400 [38]. Nested polymerase chain reaction (PCR) identified a significant percentage of clinically silent Pj colonization in 20% of non-HIV immunosuppressed patients [39]. Therefore, early detection of asymptomatic infection of Pj in blood and respiratory specimens before, and during intense immunosuppression may enable selection of cases for pre-emptive treatment of Pj infection in order to prevent the development of IRD during reversal of immunosuppression [40,41].

## Conclusions

PCP occurring in the context of IRD is not a rare phenomenon and is likely to be under-reported in the literature. In this setting, it may be more common for PCP to manifest acutely with a fulminant clinical course. Clinicians caring for immunosuppressed patients should be alert to this unique phenomenon so as to initiate timely and appropriate investigations and treatment for their patients. Serial monitoring of lymphocyte count, or if possible CD4 count, could serve as a useful adjunct to facilitate the diagnosis and management of this condition in a wide range of immunosuppressed hosts, especially in the presence of new pulmonary symptoms and/or radiographic abnormalities compatible with the diagnosis.

## Competing interests

The author(s) declare that they have no competing interests.

## Authors' contributions

RAL and DSH were involved in the clinical evaluation and treatment of patients. BST and IFH helped with literature searching and review. AKW and VCC drafted and refined the manuscript. KYY conceived the study, participated in its design and coordination, and supervised the preparation of the manuscript. All authors have read and approved the final draft of the manuscript before submission.

## Pre-publication history

The pre-publication history for this paper can be accessed here:



## Figures and Tables

**Table 1:** Summary of literature reported cases of HIV-negative immunocompromised patients with PCP manifested as IRD

Case [Ref.]	Sex/Age (years)	Underlying disease (s)	Reduction of IS level before symptoms onset of IRD	Symptoms & signs at IRD; change of lymphocyte count before & during IRD (if mentioned)	Treatment, clinical progress & outcome
1–7 [22]	M/F: 4:3 Median age 12, range 2–25	Acute leukemia in remission (4), acute leukemia in relapse (1), Hodgkin's disease (1), embryonal carcinoma of testes (1)	P ↓ from 100 mg to 40 mg over 3 weeks in 1 patients; In another 6 patients, P stopped in a median of 10.5 days, range (1–21 days) before symptoms onset	NM	Died (5) & survived (2)
8–15 [23]	NM	Primary brain tumour (8)	Dexa ↓ over a median of 12.5 days, range (1–63 days)	Fever (4), nonproductive cough (4), productive cough (2), dyspnoea (7), chest pain (4); CXR: bilateral infiltrates (3), diffuse infiltrates (3), focal infiltrates (1), clear (1)	Died (3) & survived (5)
16 [24]	M/55	Primary brain tumour (glioblastoma multiforme)	Dexa ↓ from 16 mg qd to 2 mg qd over 8 weeks	Intermittent fever, nonproductive cough, progressive dyspnoea; CXR: bilateral interstitial infiltrates; PaO_2 _(RA): 51 mmHg	Treated with intravenous cotrimoxazole; survived
17 [24]	F/74	Primary brain tumour (meningioma)	Dexa ↓ from 12 mg qd to 4 mg qd over 2 weeks	Intermittent fever, nonproductive cough; CXR: bilateral interstitial infiltrates; PaO_2 _(RA): 45 mmHg	Treated with intravenous cotrimoxazole; survived
18 [24]	M/50	Primary brain tumour (astrocytoma)	Dexa ↓ from 16 mg qd to 1 mg qd over 8 weeks	Fever, nonproductive cough, dyspnoea; CXR: bilateral interstitial infiltrates; PaO_2 _(RA): 73 mmHg	Treated with intravenous cotrimoxazole; mechanical ventilation; survived
19 [24]	M/75	Primary brain tumour (glioblastoma multiforme)	Dexa ↓ from 16 mg qd to 4 mg qd over 6 weeks	Fever, nonproductive cough, bloody diarrhoea; CXR: clear; PaO_2 _(RA): 89 mmHg	Treated with intravenous cotrimoxazole; survived
20 [25]	M/24	ACTH- producing metastatic bronchial carcinoid	Serum cortisol ↓ from 138 pg/ml to 18 pg/ml over 54 days	Fever, nonproductive cough, weakness, sweats; CXR: bilateral fluffy infiltrates; PaO_2 _(RA): 40 mmHg	Treated with intravenous cotrimoxazole; mechanical ventilation; died of malignancy
21 [26]	F/38	Endogenous Cushing's syndrome	Metyrapone 750 mg qd added 1 day before symptoms onset	Productive cough, dyspnoea; CXR: right lower upper lobe infiltrates; PaO_2 _(RA):31 mmHg	Treated with intravenous cotrimoxazole; mechanical ventilation; died
22–28 [32]	M/F 4:3 Mean (SD) age 53.1 (13.6)	ITP (2), GN (2), bullous pemphigoid (1), endogenous Cushing's syndrome (1), and renal transplantation (1)	Reduction of steroid but details of tailing regimen was not mentioned	An upsurge of lymphocyte counts from the reduction of immunosuppression (median 300/μL, range 290 to 740/μL) to the onset of IRD (median 1500/μL, range 600 to 5620/μL)	Treated with steroid as anti-PJP therapy in 7 (100%); mechanical ventilation in 6 (85.7%), died in 3 (42.9%)
29	M/33 (Our patient)	Systemic lupus erythematosus/dermato-myositis overlapping syndrome	P ↓ from 45 mg to 15 mg over 4 days	Fever, dyspnoea; CXR: increased perihilar infilitrates; lymphocyte count increased from 600 to 1300/μL	Treated with intravenous cotrimoxazole and steroid; survived

Note. Aza, azathioprine; CXR, chest radiograph; Dexa, dexamethasone; IRD, immunorestitution disease; ITP, immune thrombocytopenia purpura; IS, immunosuppression; GN, glomerulonephritis; P, prednisolone; PCP, *Pneumocystis jiroveci *pneumonia; RA, room air.

**Table 2:** Summary of literature reported cases of HIV-positive patients with IRD to PCP after HAART

Case [Ref.] Sex/Age	CD4 (/μL) & HIVRNA (log_10 _copies/ml) before HAART	Therapy of PJP & HAART regimen	Day of HAART after initiation of PCP treatment	Symptoms & signs during IRD	Day of IRD after initiation of HAART	Day of steroid withdrawal before the onset of IRD	CD4 (/μL) & HIVRNA (log_10 _copies/ml) during IRD	Therapy of IRD & clinical outcome
1 [28] M/37	7 & 5.1	Cotrimoxazole & MP; zidovudine, lamivudine, & indinavir	16 days	High fever, acute respiratory failure; CXR: patchy alveolar opacities in both upper lobes	7 days	7 days	38 & UD	Restart cotri-moxazole & stop HAART; survived
2 [28] M/47	28 & 5.0	Cotrimoxazole & MP, then aerosolized pentamidine; viramune, stavudine, & didanosine	1 day	High fever, acute respiratory failure requiring intubation; CXR: diffuse alveolar opacities	17 days	2 days	40 & 4.5	Restart MP & stop HAART; survived
3 [28] F/50	230 & 5.8	Cotrimoxazole & MP; zidovudine, lamivudine, & indinavir	16 days	High fever, acute respiratory failure; CXR: patchy alveolar opacities in both upper lobes	7 days	7 days	564 & 3.1	Start Atovaquone, aerosolized pentamidine, & steroid; survived
4–6 [29] NM	26 & 5.5 (median)	Cotrimoxazole & high dose steroid; NM	15 – 18 days (range)	Swinging fever, acute respiratory failure, & radiological deterioration	5 days (median); 3–17 days (range)	NM	62 & 2.87 (median)	Re-introducing high dose steroids & alternative PJP therapy; all three patients survived
7 [30] M/38	4 & 5.5	Atovaquone; didanosine, efavirenz, nelfinavir, & stavudine	35 days	Fever, cough, dyspnoea, & night sweats; CXR: bilateral mid & lower zone airspace shadow	14 days	NA	125 & 3.6	Intravenous pentamidine & hydrocortisone; survived
8 [30] NM	70 & NM	Cotrimoxazole; zidovudine					182 & NM	Cotrimoxazole; survived
9 [30] NM	10 & NM	Cotrimoxazole; zidovudine	21 days (median) 17–24 days (range)	Fever, dyspnoea, with or without cough	15 days (median) 5–30 days (range)	NM	30 & NM	Supportive therapy
10 [30] NM	216 & NM	Cotrimoxazole & steroid; zidovudine					340 & NM	Cotrimoxazole & steroid
11 [30] NM	290 & NM	Cotrimoxazole; zidovudine, & didanosine					430 & NM	Cotrimoxazole
12 [30] NM	60 & NM	Cotrimoxazole; zidovudine					130 & NM	Supportive therapy
13 [31] M/34	46 & > 5.9	Cotrimoxazole; zidovudine, lamivudine, lopinavir-ritonavir	18 days	Recurrent fever, chest discomfort, cough, & dyspneoa; CXR showed diffuse bilateral reticulonodular infiltrates	14 days	NA	435 & 4.5	Cotrimoxazole (pro-phylactic dose) & keeping HAART; survived

Note. CXR, chest radiograph; HAART, highly active antiretroviral therapy; IRD, immunorestitution disease; MP, methylprednisolone; NA, not applicable; NM, not mentioned; PCP, *pneumocystis jiroveci *pneumonia
